# Impact of Hospital Antimicrobial Stewardship Team on Antimicrobial Usage in Regional Clinics: A Multicenter Retrospective Study

**DOI:** 10.7759/cureus.97258

**Published:** 2025-11-19

**Authors:** Takumi Umemura, Yoshikazu Mutoh, Yuki Ito, Aiko Ota, Takahito Mizuno, Tatsuya Hioki, Tetsuya Yamada, Yoshiaki Ikeda, Toshihiko Ichihara

**Affiliations:** 1 Department of Pharmacy, Tosei General Hospital, Seto, JPN; 2 Department of Infectious Diseases, Tosei General Hospital, Seto, JPN; 3 Department of Infection and Prevention, Tosei General Hospital, Seto, JPN; 4 College of Pharmacy, Kinjo Gakuin University, Nagoya, JPN

**Keywords:** antimicrobial stewardship, antimicrobial usage, aware classification, penicillin, pharmacist

## Abstract

Background: Reports on antimicrobial stewardship teams, including hospital pharmacists, contributing to the appropriate use of antimicrobials in community clinics remain unclear. This study aimed to evaluate the impact of these series of initiatives on the appropriate use of antimicrobials in the community.

Methods: This retrospective multicenter study was conducted at community clinics belonging to the Seto-Asahi Medical Association in Japan between April 2022 and September 2024. A practice lecture about antimicrobial stewardship was started to promote community antimicrobial stewardship in November 2022 by an antimicrobial stewardship team at Tosei General Hospital. The primary outcome was the change in the monthly usage of oral penicillins, third-generation cephalosporins, quinolones, and macrolides during the study period.

Results: A total of 12 community clinics in the Seto-Asahi area were eligible. Using interrupted time series analysis, the days of therapy (DOT) for penicillins showed a statistically increased trend from period 1 (pre-promoting community antimicrobial stewardship) to period 2 (promoting community antimicrobial stewardship); the change of slope was from -0.24 to 0.13 (P = 0.03). For the DOT of third-generation cephalosporins, quinolones, and macrolides, there were no statistically significant differences in the levels or trend slopes. The DOT for the Access group increased from 2.03 (10.7%) in period 1 to 4.05 (18.9%) in period 2 (P < 0.01).

Conclusion: Promoting the appropriate use of antimicrobials through a hospital antimicrobial stewardship team in local community clinics played an important role in reducing the use of broad-spectrum antimicrobials.

## Introduction

Worsening patient prognoses due to an increase in drug-resistant bacteria have become a global threat [1]. Therefore, a global action plan in 2019 was proposed, focusing on support for optimizing the use of antimicrobial medicines, appropriate antimicrobial use, and investment in new medicines, diagnostic tools, and vaccines, to improve awareness and understanding of antimicrobial resistance (AMR), strengthen the knowledge and evidence base through surveillance and research, and reduce the incidence of infection through effective sanitation and hygiene [2]. An important element of this program was the appropriate use of antimicrobials. In hospitals, the antimicrobial stewardship team is led by physicians and pharmacists, who possess a certain level of effectiveness in improving patient prognosis and restoring the drug susceptibility of some bacteria [3-5]. However, since antimicrobial prescriptions for outpatients are higher than those for inpatients, the promotion of the appropriate use of antimicrobials for outpatients is also attracting attention [6]. Specifically, because the relative use of third-generation cephalosporins, fluoroquinolones, and macrolides is higher in Japan than that in other high-income countries, the national antimicrobial resistance action plan explicitly targets reductions in these classes.

However, in one report from 2020, only a limited number of physicians at community clinics were found to have sufficient knowledge about antimicrobial stewardship and antimicrobial resistance (AMR) action plans [7]. In addition, few reports exist on the contribution of antimicrobial use support teams, including hospital pharmacists, to the appropriate use of antimicrobials in the community, and one was published only in 2023 [8]. Tosei General Hospital is the only acute care hospital in Seto City and is responsible for acute care in the region. In our region, we initiated a series of lectures on the changes in prescribing composition consistent with stewardship goals set by hospital pharmacists from the antimicrobial stewardship team of local medical associations. This study evaluated whether community antimicrobial stewardship programs, including hospital pharmacist-led educational sessions and the presentation of facility-level antimicrobial use data from surveillance, reduced Watch class antibiotic use and increased prescriptions of Access class agents in accordance with the AWaRe (Access, Watch, Reserve) classification of the World Health Organization (WHO). Moreover, beginning in April 2024, after the intervention was launched, an additional government reimbursement under Japan’s national health insurance system was introduced for clinics in which the proportion of AWaRe-classified Access antibiotics was ≥60% or ranked within the top 30% across all clinics nationwide. To accurately attribute the effects of the intervention, antimicrobial use in each clinic was evaluated across three periods: pre-intervention, post-intervention, and post-reimbursement.

## Materials and methods

Setting

This retrospective multicenter study was conducted at Tosei General Hospital (an acute care community hospital) and at community clinics within the Seto-Asahi Medical Association in Japan that met the community clinic inclusion criteria, defined as (i) provision of antimicrobial surveillance data to Tosei General Hospital and (ii) consent to participate in community antimicrobial stewardship programs. Outcome measures were reviewed from April 2022 through September 2024. Clinics that reported monthly antimicrobial use throughout the period of interest were included; those with missing months were excluded. This study was judged not to be subject to ethical review by the Ethics Committee of Tosei General Hospital because only aggregate data, and no personal data, were used in the study (receipt number: 1338). Consent for the use of the data was obtained from the participating institutions. In November 2022, a practice was initiated to change prescribing composition consistent with stewardship goals by an antimicrobial stewardship team at Tosei General Hospital, which included one infectious disease (ID) physician and one ID pharmacist certified as an ID specialist. The dataset was aggregated from each facility’s electronic prescribing records. From December 2022 onward, the same data were extracted from the Japan Surveillance for Infection Prevention and Healthcare Epidemiology (J-SIPHE) for clinics or the Online Monitoring System for Antimicrobial Stewardship at Clinics (OASCIS), which is operated by the AMR Clinical Reference Center at the National Center for Global Health and Medicine [9].

Community antimicrobial stewardship programs

Intervention components aimed at reducing inappropriate prescribing of oral broad-spectrum antimicrobials (particularly third-generation cephalosporins, quinolones, and macrolides) and promoting the use of narrow-spectrum agents, such as penicillins, were operationalized through a total of eight quarterly lectures held every three months between April 2022 and September 2024 and delivered by ID pharmacists to the managing physicians at community clinics. During these lectures, they (1) provided an overview of Japan’s National Action Plan for appropriate antibiotic use [10,11]; (2) gave guidance on selecting antimicrobials, primarily amoxicillin, for airway bronchiolitis and acute pharyngitis in accordance with the Ministry of Health, Labour and Welfare’s Guide to the Manual of Antimicrobial Stewardship [12,13]; (3) explained the WHO AWaRe (Access, Watch, Reserve) classification and its significance [14]; and (4) presented antimicrobial use surveillance data to enable peer comparison in the Seto-Asahi area. Each presentation coincided with the eight quarterly lectures (i.e., at three-month intervals) [15]. The lectures were delivered either in person or through live webcasts. Attendance via webcast was confirmed by reviewing access logs maintained by the hosting platform.

Outcome measure

To evaluate changes in antimicrobial use attributable to the intervention, we divided the study period into the following three periods: period 1, pre-promoting community antimicrobial stewardship from April 2022 to November 2022; period 2, promoting community antimicrobial stewardship from December 2022 to March 2024; and period 3 (April 2024 to September 2024), an additional fee for the appropriate use of antimicrobials based on the WHO AWaRe classification was introduced into Japan’s medical reimbursement system and has been available to community clinics since then. Periods 2 and 3 were separated because the added reimbursement (government reimbursement to clinics under the national health insurance system) may have influenced the promotion of antimicrobial stewardship. Only facilities that submitted data for all the periods were included in the analysis. The primary outcome was the change in the monthly usage of oral penicillin, third-generation cephalosporins, quinolones, and macrolides during the study period. The antimicrobial agents included in the study were those listed in the classification scheme defined by the AMR Clinical Reference Center [16]. Additionally, changes in the use of other classes of oral drugs were examined. As a secondary outcome, the percentages of Access, Watch, and Reserve antibiotics according to the WHO AWaRe classification 2023 were examined for each period.

Data analysis

To evaluate the effect of promoting community antimicrobial stewardship, an interrupted time series analysis [4] was performed to assess changes in the days of therapy (DOT) per 100 patient visits for each class between periods 1 and 2 and periods 2 and 3. The monthly DOT per 100 patient visits was calculated as the total number of DOT for all included antimicrobials in a given month divided by the total number of outpatient patient visits in the same month, multiplied by 100. Segmented ordinary least squares (OLS) regression with Newey-West heteroskedasticity and autocorrelation (HAC) standard errors was employed to adjust for serial autocorrelation [17]. A DOT was defined as one calendar day on which a patient received a given antimicrobial, counted once per day per agent, regardless of dose or frequency; concomitant agents on the same day were counted as multiple DOTs. Kruskal-Wallis tests were performed for the DOT of all antimicrobials and the rate of Access class of AWaRe classifications during each period, and Bonferroni corrections were applied to determine statistically significant differences between groups. DOT was selected instead of defined daily doses (DDD) for the tally of antimicrobial use because DDD is based on adult dosing assumptions and thus inappropriate for datasets that include pediatric patients. Since patient-level age information was not available, using DDD could have led to biased estimates. In all analyses, two-tailed P-values < 0.05 were considered statistically significant, and analyses and figure generation were performed using IBM SPSS Statistics version 26 (IBM Corp., Armonk, NY) and R version 4.4.1 (R Foundation for Statistical Computing, Vienna, Austria).

## Results

Of the 16 community clinics that had submitted antimicrobial data as of November 2024 in the Seto-Asahi area, 12 clinics that submitted monthly data during the study period were included, and four clinics were excluded from the analysis due to missing data values, such as starting to submit data in the middle of the study. The median participation rates for the lectures were 75% (interquartile range (IQR): 71.9-78.1). The specialties and numbers of outpatients during the study period are shown in Table 1. The total DOT for oral antimicrobials (median (IQR)) in each period was as follows: period 1, 18.2 (16.7-20.0); period 2, 20.9 (18.3-23.3); and period 3, 18.7 (18.3-20.6), respectively, and there were no significant differences among all periods (P = 0.13).

**Table 1:** Background of eligible community clinics IQR: interquartile range

Clinics	Specialties	Number of outpatient visits per month during the study period, median (IQR)	
A	Internal medicine, pulmonology, pediatrics	1,424 (1,266-1,567)	
B	Internal medicine, surgery, pediatrics, gastroenterology	787 (717-832)	
C	Otolaryngology	2,921 (2,577-3,180)	
D	Internal medicine, cardiology	988 (954-1,056)	
E	Internal medicine, surgery	1,988 (1,763-2,193)	
F	Internal medicine, pediatrics, gastroenterology	986 (967-1,013)	
G	Internal medicine, pediatrics	1,178 (1,097-1,241)	
H	Internal medicine, pediatrics, cardiology	2,155 (1,991-2,392)	
I	Internal medicine, pediatrics, cardiology	1,261 (1,186-1,352)	
J	Internal medicine, surgery, pediatrics	2,458 (2,291-2,559)	
K	Internal medicine, surgery, dermatology, gastroenterology	475 (456-487)	
L	Internal medicine, surgery, dermatology, nephrology	275 (266-296)	

**Figure 1:**
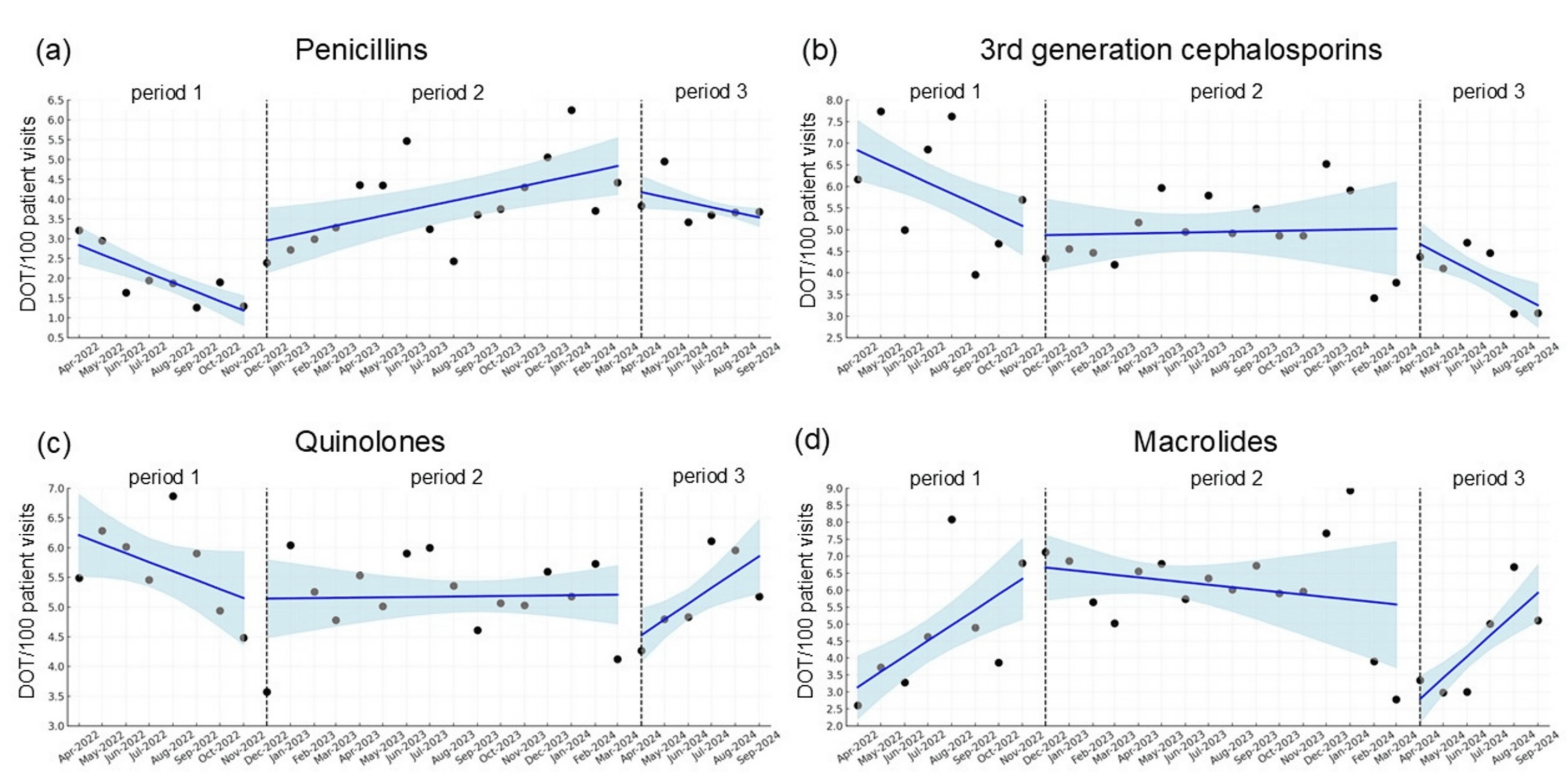
Trends in DOT for penicillins, third-generation cephalosporins, quinolones, and macrolides The explanation of each phase is as follows: period 1 (no intervention from April 2022 to November 2022), period 2 (promoting WHO Access to class antimicrobials through the hospital antimicrobial stewardship team from December 2022 to March 2024), and period 3 (after starting an additional fee for the appropriate use of antimicrobials from April 2024 to September 2024). Although no change was evident immediately after period 2 started (p = 0.13), the number of days for penicillins increased significantly (change in slope from -0.24 to 0.13, P = 0.03). There were no statistical differences in the levels or trend slopes for the other drugs between periods 1 and 2 and periods 2 and 3. DOT: days of therapy, WHO: World Health Organization

Figure 1 shows the changes in monthly observed DOT for penicillins, third-generation cephalosporins, quinolones, and macrolides across all periods. The DOT for penicillins did not statistically decrease or increase immediately after the start of period 2 (P = 0.13) but showed a significantly increasing trend over this period (change in slope from -0.24 to 0.13, P = 0.03). From period 2 to 3, there were no statistical differences in levels (P = 0.32) or trend slopes (change of slope from 0.13 to -0.13, P = 0.25). For the DOT of third-generation cephalosporins, quinolones, and macrolides, there were no statistical differences in the levels (P = 0.88, 0.08, 0.07) or trend slopes (change in slope from -0.25 to -0.01, P = 0.62; -0.15-0.004, P = 0.64; 0.46 to -0.07, P = 0.07) between periods 1 and 2. Similarly, between periods 2 and 3, there were no statistically significant differences in the levels (P = 0.62, 0.27, 0.08) or trend slopes (change in slope from -0.01 to -0.28, P = 0.16; 0.004 to 0.27, P = 0.13; and -0.07 to 0.63, P = 0.06, respectively).

**Figure 2:**
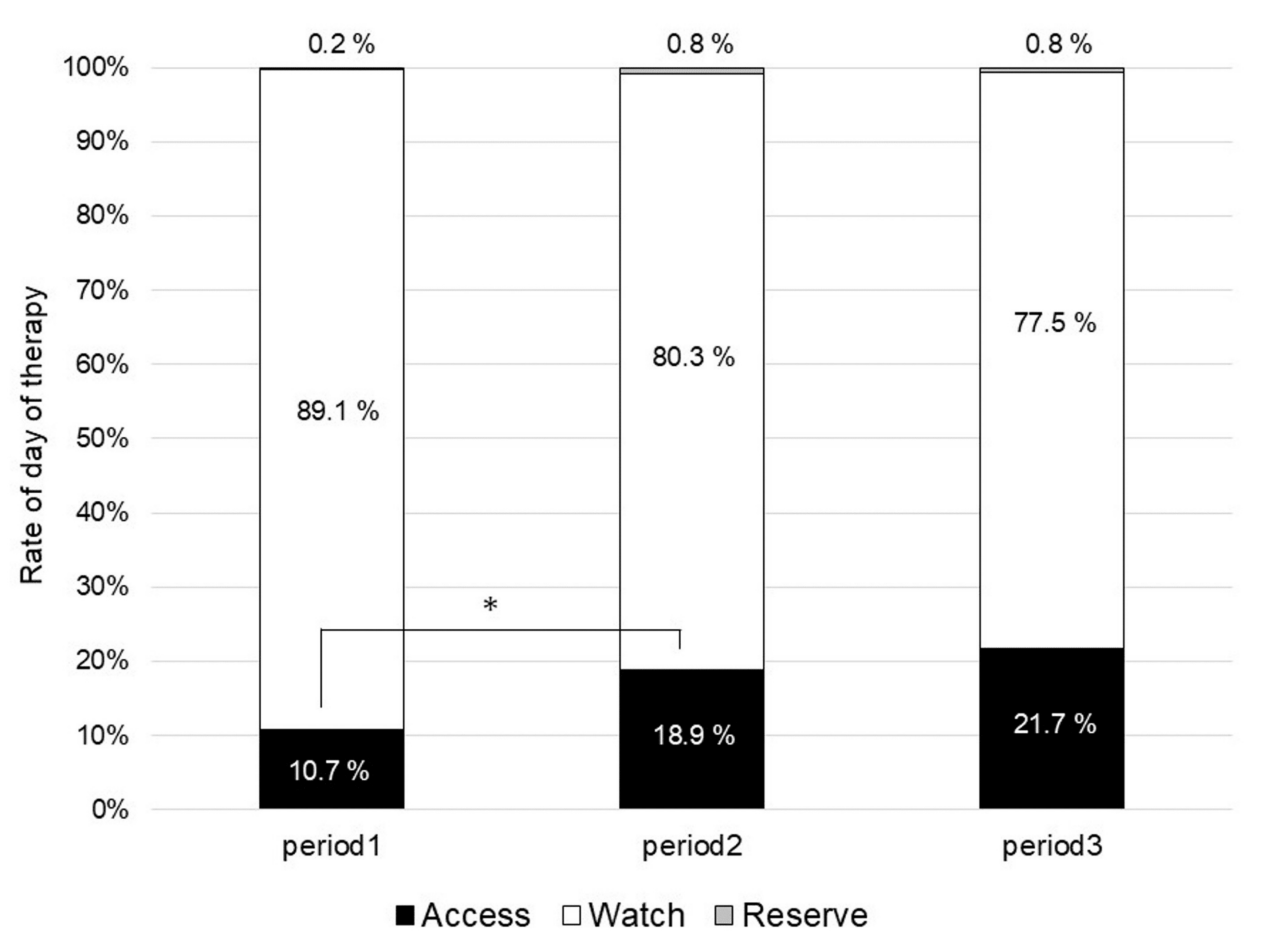
Rates of days of therapy by AWaRe classification during the study period *Between periods 1 and 2, the rate of access per month showed a statistically significant difference (P < 0.01), as indicated by the Bonferroni correction for the Kruskal-Wallis test among all periods.

The changes in DOT for the other classes are shown in the Appendices. The DOT for second-generation cephalosporins started to increase immediately after the start of period 3 (P < 0.01); however, no major increase was observed during this period (change in the slope from 0.001 to 0.006, P = 0.41). There were no statistical differences in the levels or trend slopes of the drugs between periods 1 and 2 and periods 2 and 3. Figure 2 shows the DOT rates of the AWaRe classification for each period. The DOT for Access, Watch, and Reserve were 2.03 (10.7%), 16.91 (89.1%), and 0.04 (0.2%) in period 1; 4.05 (18.9%), 17.20 (80.3%), and 0.16 (0.8%) in period 2; and 4.17 (21.7%), 14.91 (77.5%), and 0.14 (0.8%) in period 3. With the reduction in Watch antibiotics, the rate of Access per month showed a statistically significant increase between periods 1 and 2 (P < 0.01), as indicated by the Bonferroni correction for the Kruskal-Wallis test among all periods.

## Discussion

This study highlighted the critical role of hospital antimicrobial stewardship teams in improving antimicrobial use in community clinics. Our findings indicate that educational interventions by hospital antimicrobial stewardship teams were associated with access rates by promoting the use of oral penicillin without increasing the total number of days of oral antimicrobial use. Notably, this is one of the first studies to evaluate the outcomes of hospital antimicrobial stewardship teams’ interventions targeting community clinics in Japanese settings. Although some success has been reported in outpatient antimicrobial stewardship initiatives at individual facilities or by community pharmacy, studies on hospital-led efforts in community settings are scarce [18,19]. This study is the first to demonstrate intervention outcomes in community clinics that promote antimicrobial stewardship by a hospital team.

Awareness of AMR action plans is low among physicians in clinics [7]. The Manual of Antimicrobial Stewardship recommends penicillin as an antibacterial therapy in acute pharyngitis and acute sinusitis [12,13]. Routine lectures based on the Manual of Antimicrobial Stewardship have led to an increase in the rate of penicillin use, a decrease in the use of third-generation cephalosporins and quinolones, and a decrease in the upward trend in macrolide use. As a result, the rate of Access increased, and the rate of Watches decreased. Mechanistically, our findings suggest that lectures delivered by pharmacists with ID expertise facilitate knowledge transfer, while regular feedback on facility-level antimicrobial use fosters feedback accountability, together plausibly inducing changes in prescribing behavior. The use of antimicrobials varies depending on the prevalence of IDs. In Aichi Prefecture, group A streptococcal pharyngitis, in which penicillin drugs were actively used, continued to be prevalent from late October 2023 to August 2024 [20]. As our survey showed an upward trend in penicillin use before the pharyngitis epidemic, we believe that our continued lectures will have increased the use of penicillin products and further accelerated their use. In addition, the nationwide supply restriction of penicillin from July 2023 threatened to reduce the proportion of prescriptions in Japan [21]; however, continuous efforts to maintain the use of penicillin prevented a reduction in the proportion of prescriptions.

Furthermore, an additional fee for the appropriate use of antimicrobials became available in 2024 in Japan’s medical reimbursement system. The evaluation was divided into three periods to account for the possibility of changes in antimicrobial use due to the impact of the additional fee. The results revealed that the support of the hospital’s antimicrobial stewardship team could change antimicrobial use even before the implementation of the additional fee system. This indicates that not only additional financial incentives but also educational activities by hospital antimicrobial stewardship teams on the proper use of antimicrobial agents are important factors in changing physicians’ prescription behaviors. Inappropriate prescription of oral broad-spectrum antimicrobials contributes to the increase of drug-resistant bacteria and leads to adverse events such as *Clostridioides difficile* infection, which requires appropriate selection of antimicrobials for use, for example, choosing penicillins for acute sinusitis [22]. We believe that our intervention will help to increase the use of penicillins. Using a large reimbursement database, Jindai et al. concluded that while there was a short-term reduction in oral antimicrobial prescriptions, there was no long-term trend [8]. Although our study did not investigate the extent of inappropriate use, it did indicate that more detailed support beyond lectures is needed to lead to continued behavioral changes among clinical physicians to enable them to refrain from prescribing antimicrobials. Further, patients or their families may request antimicrobial prescriptions, as experienced by approximately half the physicians in the study [7]. Therefore, patient education is also necessary to reduce prescriptions.

This study had some limitations. First, the outcome of appropriate antimicrobial use in the AMR action plan is the recovery or prevention of bacterial susceptibility to drugs by reducing inappropriate broad-spectrum antimicrobial use. As most of the clinics surveyed in this study did not conduct microbiological testing, we were unable to evaluate the changes in such outcomes.

Second, the evaluation covered a limited set of 12 clinics within a single regional medical association, capturing only a portion of outpatient care in the area and not necessarily representing overall regional antimicrobial use. Because healthcare delivery models may differ across other prefectures in Japan and in other countries, the findings of this study beyond the study area should be interpreted with caution.

Third, our analysis did not explicitly account for contextual factors that may modify the intervention’s effectiveness, including population demographics, clinic size, antibiotic availability, the lack of a control group, the potential for residual confounding, and concurrent public health campaigns. Because these data were not consistently available across clinics and time, we could not incorporate them into the segmented time series models. This limitation may partly explain heterogeneity in prescribing responses across facilities. Future work should incorporate these contextual elements, where feasible, through stratified analyses or models adjusted for facility- and population-level covariates. In addition, at least one managing physician at each facility had attended the lecture, but not all did, and the percentage of physicians who attended the lecture was unknown.

Fourth, because the study did not specify clinical departments or perform disease-specific analyses, we could not delineate the indications for which antimicrobials were prescribed; likewise, we did not conduct detailed analyses of infection cure rates, resistance patterns, or patient satisfaction. The study included data before the use of OASCIS, and these were not tabulated for disease names; therefore, a more detailed analysis is desirable in the future.

Finally, the absence of an external control group or a counterfactual trend from similar non-intervention clinics limits causal inference. Consequently, the observed reductions in antimicrobial use may partially reflect broader secular trends, seasonal fluctuations, or nationwide awareness campaigns occurring during the study period. Moreover, we acknowledge that seasonal variation was not incorporated into the present models, primarily because the observation windows, most notably in the final segment, were relatively short. This omission may attenuate or obscure any true seasonal patterns and should be interpreted as a limitation of the study.

## Conclusions

This multicenter study demonstrated the positive effects of hospital antimicrobial stewardship team support on oral antimicrobial use in community clinics. Educational interventions were associated with increasing penicillin use, improved access rates, and changes in prescribing behavior without increasing total antimicrobial use. These findings highlight the pivotal role of hospital antimicrobial stewardship teams in promoting antimicrobial stewardship in the community.

## Figures and Tables

**Figure 3:**
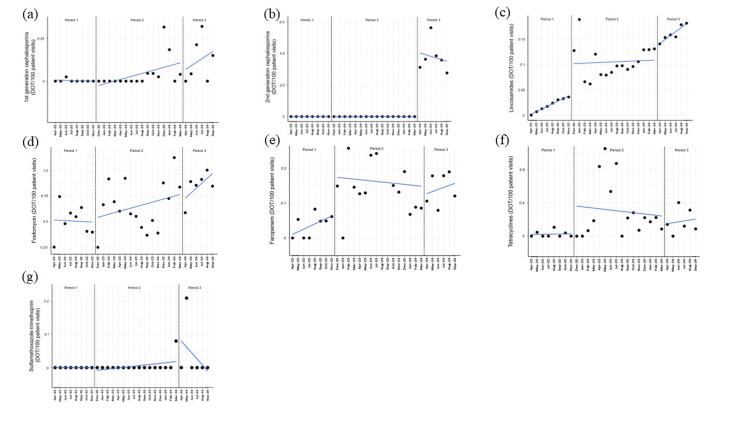
Trends in DOT for first-generation cephalosporins, second-generation cephalosporins, lincosamides, fosfomycin, faropenem, tetracyclines, and sulfamethoxazole-trimethoprim by month during the study periods, with slopes based on linear regression in each period (a) DOT for first-generation cephalosporins, (b) DOT for second-generation cephalosporins, (c) DOT for lincosamides, (d) DOT for fosfomycin, (e) DOT for faropenem, (f) DOT for tetracyclines, and (g) DOT for sulfamethoxazole-trimethoprim DOT: days of therapy

## Appendices

Figure 3 and Table 2 show the changes in DOT for first-generation cephalosporins, second-generation cephalosporins, lincosamides, fosfomycin, faropenem, tetracyclines, and sulfamethoxazole-trimethoprim. 

**Table 2:** Results of interrupted time series analysis of DOT for first-generation cephalosporins, second-generation cephalosporins, lincosamides, fosfomycin, faropenem, tetracyclines, and sulfamethoxazole-trimethoprim by month during the study periods, with slopes based on linear regression in each period The DOT of second-generation cephalosporins started to increase immediately after the start of period 3 (P < 0.01), but a major increase was not observed during this period (change of slope from 0.001 to 0.006, P = 0.41). There were no statistical differences in levels or trend slopes for the drugs between periods 1 and 2, and periods 2 and 3. DOT: days of therapy

Drug or class	Period 1-2			Period 2-3		
	P-value for level change	Slope change	P-value for slope change	P-value for level change	Slope change	P-value for slope change
First-generation cephalosporins	0.82	-0.001 to 0.001	0.89	0.59	0.001 to 0.021	0.61
Second-generation cephalosporins	0.89	0.000 to 0.001	0.33	<0.01	0.001 to 0.006	0.41
Lincosamides	0.47	0.005 to 0.001	0.48	0.23	0.001 to 0.008	0.31
Fosfomycin	0.33	-0.003 to 0.015	0.63	0.79	0.015 to 0.048	0.53
Faropenem	0.64	0.008 to -0.002	0.55	0.39	-0.002 to 0.006	0.27
Tetracyclines	0.68	0.001 to -0.008	0.86	0.77	-0.008 to 0.011	0.81
Sulfamethoxazole-trimethoprim	0.79	0.000 to 0.009	0.65	0.15	0.009 to -0.018	0.10
